# Double Xp11.22 deletion including *SHROOM4* and *CLCN5* associated with severe psychomotor retardation and Dent disease

**DOI:** 10.1186/s13039-015-0107-x

**Published:** 2015-02-01

**Authors:** Narjes Armanet, Corinne Metay, Sophie Brisset, Georges Deschenes, Dominique Pineau, François M Petit, Federico Di Rocco, Michel Goossens, Gérard Tachdjian, Philippe Labrune, Lucie Tosca

**Affiliations:** Service d’Histologie, Embryologie et Cytogénétique, Hôpitaux Universitaires Paris-Sud. Hôpital Antoine Béclère, 157 rue de la Porte de Trivaux, 92141, Clamart, F-92140 France; Université Paris-Sud, Le Kremlin-Bicêtre, F-94276 France; Plateforme de Génomique IMRB 955, Hôpital Henri Mondor, Créteil, F-94010 France; Service de Néphrologie pédiatrique, Hôpital Robert Debré, Paris, F-75935 France; Laboratoire de Génétique Moléculaire, Hôpitaux Universitaires Paris-Sud. Hôpital Antoine Béclère, Clamart, F-92140 France; Service de Neurochirurgie pédiatrique, Hôpital Necker Enfants Malades, Clamart, F-75015 France; Université Paris Est, Créteil, F-94010 France; Service de Pédiatrie, Hôpitaux Universitaires Paris-Sud. Hôpital Antoine Béclère, Clamart, F-92140 France

**Keywords:** Deletion, Xp11.22, Array-comparative genomic hybridization, Renal proximal tubulopathy, Dent disease, *SHROOM4*, *CLCN5*

## Abstract

**Background:**

Here we report the clinical and molecular characterization of two Xp11.22 deletions including *SHROOM4* and *CLCN5* genes. These deletions appeared in the same X chromosome of the same patient.

**Results:**

The patient is a six-year-old boy who presented hydrocephalus, severe psychomotor and growth retardation, facial dysmorphism and renal proximal tubulopathy associated with low-molecular-weight proteinuria, hypercalciuria, hyperaminoaciduria, hypophosphatemia and hyperuricemia. Standard and high resolution karyotypes showed a 46,XY formula. Array-CGH revealed two consecutive cryptic deletions in the region Xp11.22, measuring respectively 148 Kb and 2.6 Mb. The two deletions were inherited from the asymptomatic mother.

**Conclusions:**

Array-CGH allowed us to determine candidate genes in the deleted region. The disruption and partial loss of *CLCN5* confirmed the diagnostic of Dent disease for this patient. Moreover, the previously described involvement of *SHROOM4* in neuronal development is discussed.

## Background

X-linked transmission is a particular mode of transmission as the X chromosome is present as two copies in females but only as one copy in males. Thus, the consequences of X chromosome anomaly are much more common in males than females. Intellectual disability (ID) is defined as a global deficiency in cognitive function (IQ < 70) with an onset during childhood and associated with a diminished ability to adapt to the daily demands of a normal social environment. ID occurs in 1-3% of the general population [1]. Over 10-12% of all cases of ID are thought to be X-linked (XLMR) [2,3], which can be subdivided into syndromic forms (S-XLMR), and non-syndromic forms (NS-XLMR). It is known that up to 100 X-chromosomal genes could contribute to NS-XLMR many of them still unknown. [4-6]. Indeed, more than 300 X-linked protein-coding genes are expressed in brain tissue, suggesting that many XLMR genes remain to be identified [3]. Interstitial deletion within the short arm of X chromosome is an uncommon chromosome structural abnormality that has been studied essentially by conventional cytogenetic techniques.

So far, 11 cases of Xp11.22 deletions concerning 4 families have been described [7-10]. These patients had a unique deletion, often associated with autism spectrum disorders [7]. None of them have been described with Dent disease (OMIM number #300009) which is an X-linked inherited renal condition characterized by proteinuria, hypercalciuria and hyperphosphaturia, kidney stones, and in some cases, kidney failure [11-13]. Here we report the first clinical description and molecular characterization using array-CGH of a patient presenting two maternally inherited interstitial deletions of Xp11.22 in the same chromosome associated with severe psychomotor retardation and Dent disease.

## Case presentation

**Figure 1:**
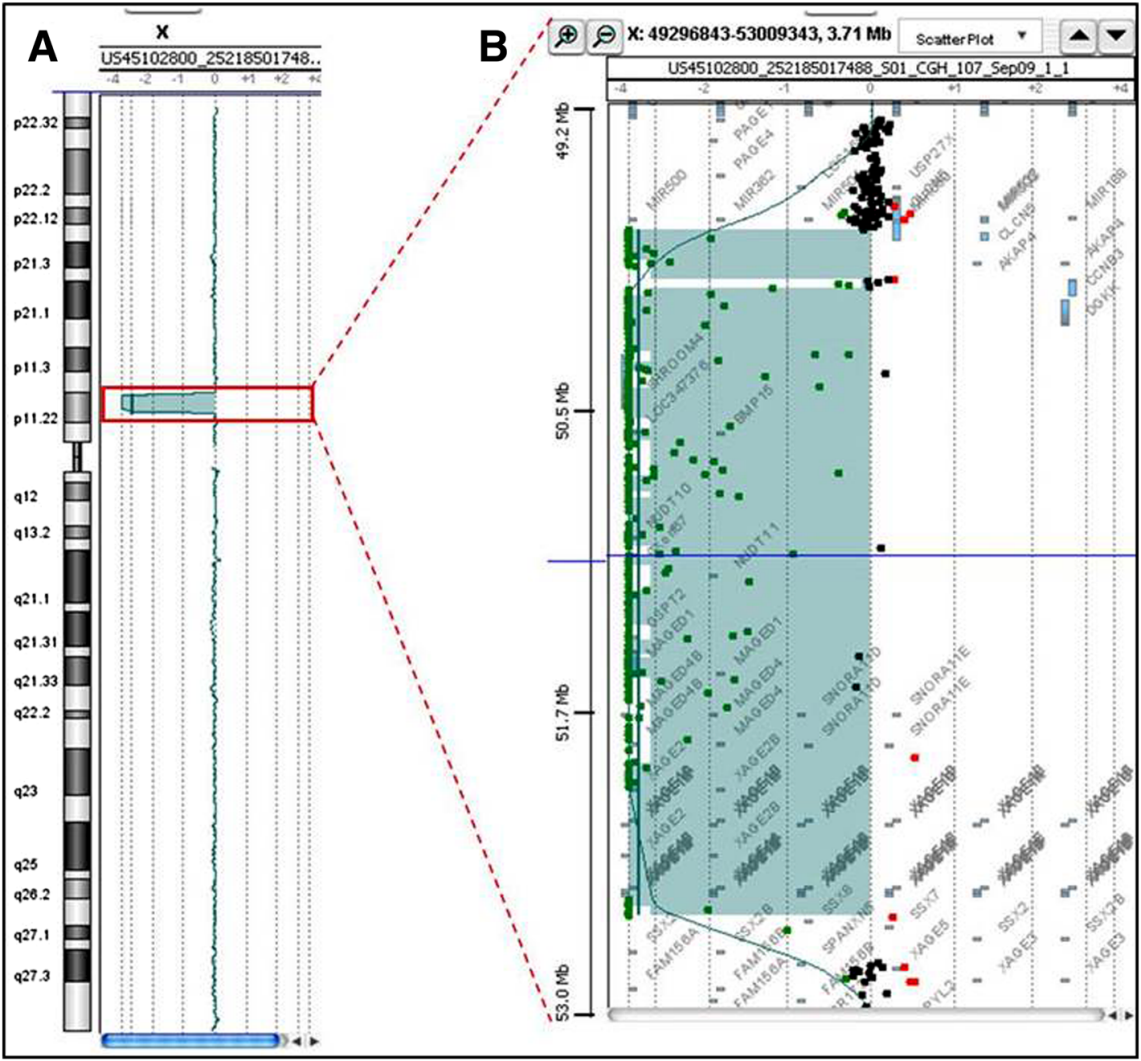
**Chromosome X profile from array-CGH with whole chromosome (A) and the two consecutive and interstitial Xp11.22 deletions (B).**

**Figure 2:**
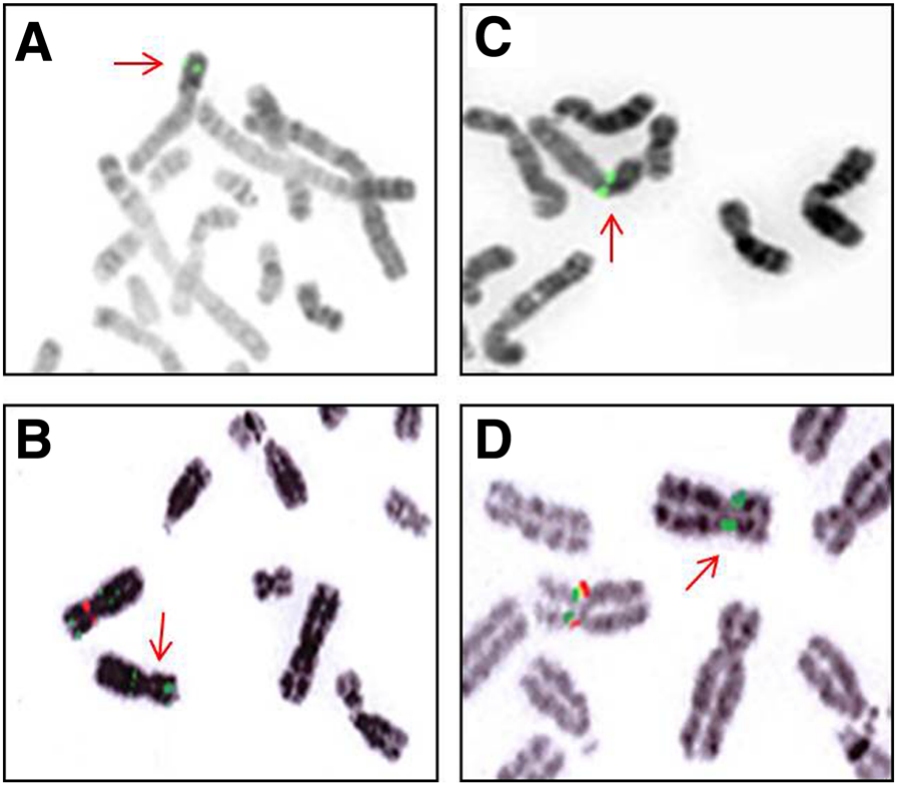
**Evidence on FISH analysis of the Xp11.22 deletions in the patient (A, C) and his mother (B, D) lymphocytes attested by the absence of one red signal on metaphase spread for the BAC clone RP3-519 N18 for the first deletion (control BAC clone RP11-122 F2 in green) and for the BAC clone RP11-637B23 for the second deletion (control BAC clone RP11-258C19 in green).**

Standard and high resolution karyotypes on cultured peripheral lymphocytes showed a 46,XY formula for the patient. FISH analyses of subtelomeric regions were normal. The use of array-CGH (180 K) revealed two consecutive deletions in the region Xp11.22, measuring respectively 148 Kb and 2.6 Mb and including 2 genes for the first deletion and 23 genes for the second deletion (Figure 1A, B and Table 1). These results were confirmed with a 244 K analysis. Analyses did not reveal other significant variations (gain or loss) on the other chromosomes. BAC clones RP11-519 N18 and RP11-637B23 did not give any signal on chromosome X while BAC clones RP11-122 F2 and RP11-258C19 gave a signal on chromosome X (Figure 2A, C). The family investigation showed that the two deletions were inherited from the mother, asymptomatic (Figure 2B, D).

**Table 1:** **Both deletions, genomic positions and genes concerned based on hg19 (GRCh37)**

**Chromosome region**	**Genomic position**	**Length**	**Count of gene symbols**	**Gene Symbols**
Xp11.22	49,823,986-49,971,921	147936	2	*AKAP4, CLCN5*
Xp11.22	50,070,457-52,693,963	2623507	23	*BMP15, CCNB3, CENPVP1, CENPVP2, DGKK, GSPT2, MAGED1, MAGED4, MAGED4B, NUDT10, NUDT11, SHROOM4, SNORA11D, SNORA11E, SSX7, SSX8, XAxGE1A, XAGE1B, XAGE1C, XAGE1D, XAGE1E, XAGE2, XAGE2B*

Concerning the couple’s second pregnancy, fetal cytogenetic analysis performed on trophoblast cells at 12 weeks of gestation showed a 46,XY formula. FISH analysis showed that the male fetus had also inherited the two maternal deletions from the mother. According to the French law, the parents decided to terminate the pregnancy. No autopsy was performed.

Concerning the third pregnancy, fetal sex was determined on maternal blood sample at 11 weeks of gestation, and shown to be female. Ultrasound examinations were performed at 12, 22 and 32 weeks of gestation and did not show any abnormality. A girl was vaginally delivered at 39 weeks, and the neonatal period was uneventful. This girl is now 15 months old and is growing normally. She has been able to walk at 13 months. Unfortunately, the girl was not available for further cytogenetic explorations.

Taking into consideration that the asymptomatic mother carried the aberrant X chromosome inherited by the proband and by the male fetus aborted upon a subsequent pregnancy, X-inactivation assay was performed on the mother by analyzing the human androgen receptor CAG repeat polymorphism [14]. Results showed that she was homozygote for the androgen receptor polymorphism so we were not able to conclude on her X-inactivation pattern.

## Conclusions

Here we report two familial interstitial deletions of Xp11.22 in the same X chromosome of the same patient presenting with hydrocephalus, severe growth and psychomotor retardation, renal proximal tubulopathy with low-molecular-weight proteinuria, hypercalciuria, hyperaminoaciduria, hypophosphatemia and hyperuricemia. Maternal transmission of the deletions was also confirmed in a second fetus who was aborted due to the causality of the deletions.

So far, 11 cases of Xp11.22 deletions in 4 families have been described [7-10]. The patients’ deletions involving this region presented a unique deletion often associated with autism spectrum disorders, variable degrees of ID, craniofacial dysmorphism, long hands and fingers, normal growth parameters and significant language delay [7]. The genomic positions of these previously described deletions did not overlap with ours. Two other cases described in DECIPHER database concerned deletions that were much smaller than our patient’s deletions. These patients presented ID and short seizures. Thus, to our knowledge, this is the first case of two deletions in the same chromosomal region Xp11.22 associated with severe psychomotor retardation and renal tubulopathy.

Among the genes partially or totally included in the deletions, *SHROOM4* and *CLCN5* are candidate genes whose total loss of expression could explain clinical manifestations. Severe ID is one of the major sign that presented our patient. In 2006, Hagens et al., investigated the breakpoints of two balanced X;autosome translocations in two unrelated female patients with mild/moderate syndromic ID and found that the Xp11.2 breakpoints disrupt *SHROOM4* in both cases [15]. They also identified a missense exchange in this gene, segregating with the Stocco dos Santos XLMR syndrome in a large four-generation pedigree but absent in more than 1000 control X-chromosomes. Among other phenotypic characteristics, the affected males in this family presented with severe ID, delayed or no speech, seizures and hyperactivity.

Yoder and Hildebrand described the characterization of mouse *SHROOM4* and observed that the protein localizes to a distinct population of F-actin and appears to regulate the formation of this cytoskeletal compartment [16]. Myosin II is also localized to this compartment and appears to be required for maintaining its proper organization. Thus, *SHROOM4* encodes a protein involved in cytoskeletal architecture and may function to regulate cellular and cytoskeletal architecture by modulating the spatial distribution of myosin II [16]. *SHROOM4* is also expressed in human adult and fetal brain structures and is needed to establish or maintain a particular cytoskeletal structure that is necessary for the function or survival of a specific neuronal population of cells [16]. These data suggest that *SHROOM4* is important in cognitive function and/or development. *SHROOM4* is included in the distal Xp11.22 deletion of our patient. The total loss of function could be responsible for his phenotype. Screening of many more XLMR patients is also warranted to further confirm the implication of this gene in XLMR. This could include cellular properties such as morphology, vesicular trafficking or polarity, all of which are regulated by the actin cytoskeleton. Based on these observations, it will be important to determine what other developmental processes might be regulated by *SHROOM4* and if there are genetic interactions between *SHROOM4* and other regulators of cellular morphology.

Others genes could be potentially candidate for the ID of the patient. Indeed, western blot analysis of *MAGED1* detected expression in embryos, especially in cerebral cortex and cerebellum. *MAGED1* plays important roles in the central nervous system in both developmental and adult stages [17].

*CLCN5* (chloride channel 5) is located on chromosome Xp11.22 and encodes CIC-5, member of the CIC family (chloride ion channels and ion transporters). CIC-5 is a Cl-/H+ exchanger that is primarily localized to endosomal membranes and may function to facilitate reabsorption by the renal proximal tubule [18,19]. Mutations in *CLCN5* have been found in Dent disease, an X-linked inherited renal condition characterized by proteinuria, hypercalciuria and hyperphosphaturia, kidney stones, and in some cases, kidney failure [11-13].

To date, mutations in the gene are described and alternatively spliced transcript variants encoding different isoforms have been found. The total number of reported *CLCN5* mutations is 148, and these are scattered throughout the coding region, with no evidence for major mutational hot spots [20]. The majority of the mutations are predicted to result in truncated or absent ClC-5 protein, which would lead to complete loss of Cl-/H+ exchanger function. There is genetic heterogeneity for Dent disease, with approximately 60% of patients having *CLCN5* mutations (Dent disease 1), ~15% harboring *OCRL1* mutations (Dent disease 2) and the remaining 25% of patients having neither *CLCN5* nor *OCRL1* mutations but possibly defects in other genes [21]. The possibility that these other genes may encode some of the proteins (e.g. ClC-4 and cofilin) that interact with ClC-5 [22] has been investigated but no mutations in *CLCN4* or *COFILIN* were identified yet [20].

Besides the gene content of the two interstitial deletions and the most plausible candidates, one may not exclude position effects on neighboring genes which are not included in the deletions.

To our knowledge, this patient is the first description of a double Xp11.22 deletion causing a *CLCN5* disruption and associated with a such characteristic renal proximal tubulopathy. The diagnosis of Dent disease is usefully based on the presence of all three of the following criteria: low-molecular-weight proteinuria, hypercalciuria and at least one of the following: nephrocalcinosis, kidney stones, hematuria, hypophosphatemia or renal insufficiency. Generally, molecular genetic testing and the presence of a *CLCN5* mutation confirm the diagnosis. For our patient, the Xp11.22 deletion including *CLCN5* and, consequently, the loss of CIC-5 exchanger allowed us to confirm the diagnosis of Dent disease. The genotype-phenotype correlation was discussed with the family during genetic counseling. Prenatal diagnosis was proposed for the second pregnancy and the couple decided, according to the French law, to terminate the pregnancy with an affected male.

All findings strongly implicate the inherited Xp11.22 deletions as causative of the described somatic and developmental phenotypes in the affected male patient. The Xp11.22 deletions with loss of *CLCN5* gene allowed us to confirm the diagnostic of Dent disease for our patient. Our results also strengthen the potential role of *SHROOM4* in syndromic XLMR.

## Methods

### Patient data

**Figure 3:**
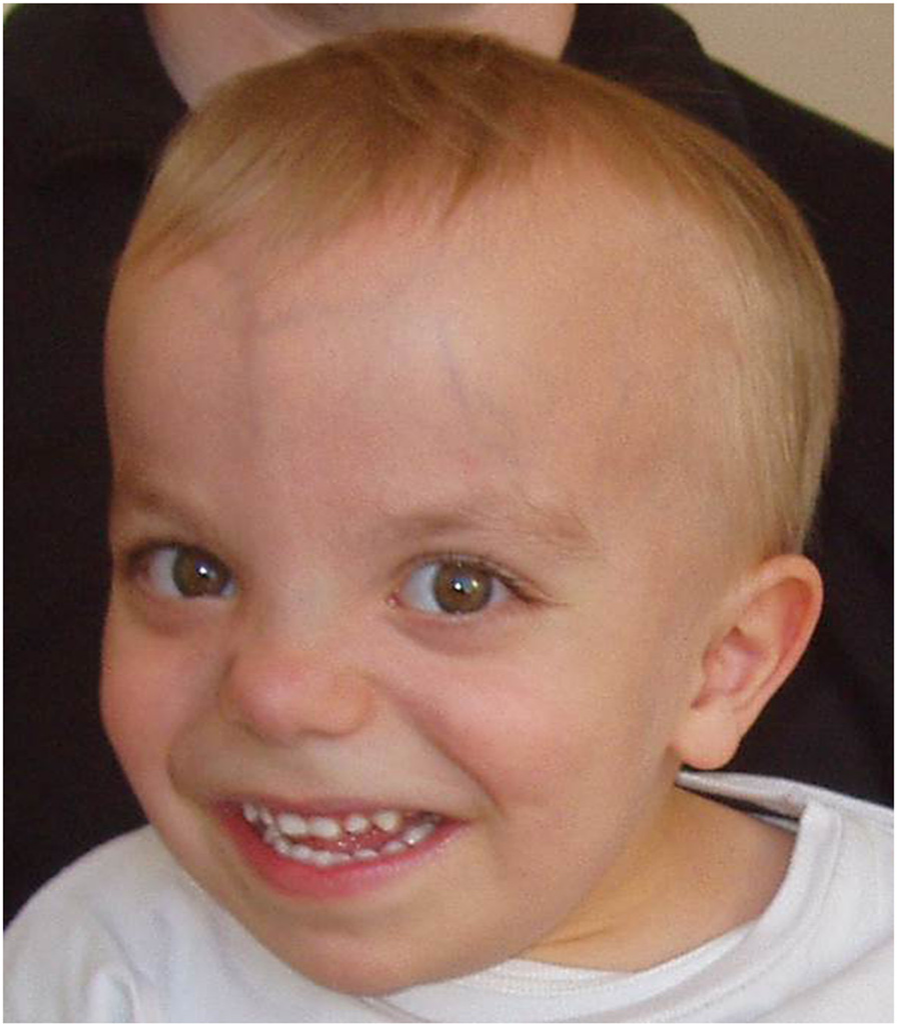
**Photograph of the patient at 4 years underlying facial dysmorphy.** Note prominent philtrum, large forehead, retrognathia, thin upper lip and low-set ears.

The patient was the product of the first pregnancy of healthy and non-consanguineous parents. During this first pregnancy the first trimester ultrasound showed an increased nuchal translucency (3.2 mm for a 63.1 mm cranio-caudal length). Prenatal cytogenetic analysis performed on trophoblast cells at 12 weeks of gestation showed a normal male karyotype. Ultrasound examinations at 18 and 22 weeks of gestation showed recurring polyhydramnios, and the amniotic fluid has been evacuated three times. A premature membrane rupture occurred at 33 weeks and 2 days of gestation and the child born by cesarean section due to a breech presentation. Height, weight, and head circumference were 2280 g (55th percentile), 43 cm (30th percentile), and 33 cm (90th percentile), respectively. The neonate was found to be mildly hypotonic. During the first 24 hours, polyuria (5 ml/kg/h) and glycosuria were noted, leading to a weight loss of 15%. Peripheral infusion was inserted for three days until the infant was able to be bottle fed. The infant was discharged at 3 weeks. Since the age of 7 months, this child had hydrocephalus, which required ventriculocysternostomy at the age of one year (stenosis of the acqueduct of Sylvius), a major growth retardation (−4SD for size and weight) and an overall severe psychomotor retardation. Walking was acquired at 4.5 years and language was reduced to a few disyllabic words. At the age of four-year-old, this patient was referred for evaluation of severe growth retardation associated with both motor and cognitive delays. The child presented a prominent philtrum, large forehead, retrognathia, thin upper lip and low-set ears (Figure 3). Moreover, the child presented renal proximal tubulopathy with low-molecular-weight proteinuria, hypercalciuria, hyperaminoaciduria, hypophosphatemia and hyperuricemia, as observed in Dent disease.

During the couple’s second pregnancy, the first trimester ultrasound examination showed an increased nuchal translucency (4,5 mm for a 58.3 mm cranio-caudal length). After fetal cytogenetic analysis and according to the French law, the parents decided to terminate the pregnancy.

During the third pregnancy, fetal sex was determined on maternal blood sample at 11 weeks of gestation, and shown to be female. Ultrasound examinations were performed at 12, 22 and 32 weeks of gestation and did not show any abnormality. A girl was vaginally delivered at 39 weeks, and the neonatal period was uneventful.

Parents showed a normal phenotype and no particular personal history.

### Conventional cytogenetic and Fluorescent *in Situ* Hybridization (FISH) experiments

Standard and high resolution chromosome analyses were performed from trophoblast cells and cultured peripheral lymphocytes of the patient using standard procedures (G-band by trypsin using Giemsa (GTG), R-band after heat denaturation and Giemsa (RHG) banding techniques and Giemsa staining). Fluorescent *in sit*u hybridization (FISH) analyses were performed on lymphocyte metaphase spreads of the patient, his parents and his maternal grandmother. The following probes were used according to manufacturer’s recommendations: a subtelomeric probe specific for all chromosomes (Vysis, Downers Grove, USA). Bacterial artificial chromosome (BAC) clones specific for the Xp11.22 chromosomal region were used to confirm the deletions (RP3-519 N18, RP11-637B23) and BAC clones were chosen from the outside of deleted regions (RP11-258C19, RP11-122 F2) (Bluegnome, Cambridge, UK).

### Oligonucleotide based array comparative genomic hybridization analysis (array-CGH)

Genomic DNA of the patient was isolated from peripheral blood using a DNeasy Blood and Tissue Kit (Qiagen, Courtaboeuf, France). The extracted DNA concentration was estimated using a NanoDrop ND-1000 spectrophotometer (NanoDrop Technologies, Wilmington, DE, USA). Genomic imbalances were analyzed by array-CGH using 180 K and 244 K oligonucleotide arrays (HU-180 K, Hu-244A, Agilent Technologies, Massy, France). Hybridization was performed according to the manufacturer’s recommended protocol and as previously described. Captured images were processed with Feature Extraction software (10.7.3.1) and data analysis was performed with Genomic Workbench V5.0.14 (Agilent Technologies). The genomic positions were determined using the version 19 of the Human Genome (http://genome.ucsc.edu/). The ADM2 algorithm was used for statistical analysis. Copy number variations (CNV) were considered significant if they were defined by three or more oligonucleotides and spanned at least 40 Kb, contained at least one gene and were not identified in the Database of Genomic Variants at the Centre for Applied Genomics (http://projects.tcag.ca/cgi-bin/variation/gbrowse/hg18/). The Nexus Copy Number Standard edition software (Proteigene, Saint-Marcel, France) algorithm was used for statistical analysis, with build 19 of the human genome (http://genome.ucsc.edu/).

### X-inactivation molecular assay

2 μg of native DNA was digested by HpaII FastDigest enzyme (NewEngland BioLabs, Evry, France). The polymorphic locus into the androgen receptor gene (CAG-repeat) was PCR-amplified with 6-FAM tagged primers from both undigested and digested DNA. PCR products were then separated onto ABI Prism3130 genetic analyzer (Applied Biosystems, Courtaboeuf, France). The PCR fragment size and area under the curve were determined in comparison with the internal size standard GS500LIZ (Applied Biosystems) by GeneMapper software (Applied Biosystems). X-chromosome inactivation ratio (XCR) was calculated according to the formulae: (AUC-1_D_/AUC-1_ND_)/((AUC-1_D_/AUC-1_ND_) + (AUC-2_D_/AUC-2_ND_)) for allele 1 and (AUC-2_D_/AUC-2_ND_)/((AUC-1_D_/AUC-1_ND_) + (AUC-2_D_/AUC-2_ND_)) for allele 2 with AUC-1, area under the curve for allele 1; AUC-2, area under the curve for allele 2; D, digested; and ND, undigested. XCR was considered as skewed over 80/20 and highly skewed over 90/10.

Experimental research on patients was done according to French law (R.162-16-7 and R.145-15-4).

## Consent

Written informed consent was obtained from the patient's parents for the publication of this report and any accompanying images.
